# Identification and characterization of tumor and stromal derived liquid biopsy analytes in pancreatic ductal adenocarcinoma

**DOI:** 10.1186/s13046-024-03262-x

**Published:** 2025-01-16

**Authors:** Julian Götze, Kira Meißner, Thais Pereira-Veiga, Yassine Belloum, Svenja Schneegans, Jolanthe Kropidlowski, Joao Gorgulho, Alina Busch, Kim Christin Honselmann, Martin Schönrock, Arne Putscher, Sven Peine, Christine Nitschke, Ronald Simon, Volker Spindler, Jakob Robert Izbicki, Thilo Hackert, Carsten Bokemeyer, Klaus Pantel, Faik Güntaç Uzunoglu, Marianne Sinn, Harriet Wikman

**Affiliations:** 1 Department of Tumor Biology, Center of Experimental Medicine, University Medical Center Hamburg-Eppendorf, Martinistr, 52, 20248 Hamburg, Germany; 2 Department of Oncology, Hematology and Bone Marrow Transplantation With Section Pneumology, University Cancer Center Hamburg, Martinistr, 52, 20248 Hamburg, Germany; 3 Department of General, Visceral and Thoracic Surgery, University Medical Center Hamburg-Eppendorf, Hamburg, Germany; 4 Department of Surgery, University Medical Center Schleswig-Holstein, Campus Luebeck, Luebeck, Germany; 5 Department of Transfusion Medicine, University Medical Center Hamburg-Eppendorf, Hamburg, Germany; 6 Mildred Scheel Cancer Career Center, University Medical Center Hamburg-Eppendorf, Hamburg, Germany; 7 Department of Pathology, University Medical Center Hamburg-Eppendorf, Hamburg, Germany; 8 Institute of Anatomy and Experimental Morphology, University Medical Center Hamburg-Eppendorf, Hamburg, Germany

**Keywords:** PDAC, CCAFs, Liquid biopsy, Stroma, Hyaluronan

## Abstract

**Background:**

The lack of predictive biomarkers contributes notably to the poor outcomes of patients with pancreatic ductal adenocarcinoma (PDAC). Cancer-associated fibroblasts (CAFs) are the key components of the prominent PDAC stroma. Data on clinical relevance of CAFs entering the bloodstream, known as circulating CAFs (cCAFs) are scarce. Here, we developed a combined liquid biopsy assay to detect cCAFs and circulating tumor cells (CTCs) in metastatic PDAC (mPDAC) and other metastatic gastrointestinal malignancies (mGI). In addition, we evaluated plasma hyaluronan (HA) levels as a complementary surrogate biomarker of the stromal extent in patients with PDAC.

**Methods:**

A sequential liquid biopsy assay based on a two step-enrichment, combining marker dependent and independent cell enrichment, was established for cCAF and CTC detection and validated in mPDAC and mGI patients. The enriched cells were identified by multiplex immunofluorescence. HA measurement was performed by ELISA on blood samples from healthy blood donors (HD), localized and late-stage PDAC patients.

**Results:**

cCAFs (≥ 1cCAFs/7.5 mL blood) were detected in 95.4% of mPDAC and in 78.2% of mGI patients, with significantly higher numbers in mPDAC compared to mGI patients (mean number 22.7 vs. 11.0; *P* = 0.0318). mPDAC patients with ≥ 15 cCAFs/7.5 mL blood had a significant shorter median overall survival (mOS 3.2 months (95% confidence interval (CI) 0.801–5.855) vs. 14.2 months (95% CI 6.055–22.332); *P* = 0.013), whereby CTC levels were not associated with mOS. In mGI neither cCAFs nor CTCs had a significant impact on OS. HA plasma levels in mPDAC patients were significantly higher compared to HD (mean 123.0 ng/mL vs. 74.45 ng/mL, *P* = 0.015). High HA in localized and late-stage PDAC were associated with a significantly shorter mOS (mOS_localized PDAC_: 12.6 months vs. 23.5 months (*P* = 0.008); mOS_mPDAC_: 1.8 months vs. 5.3 months (*P* = 0.004)).

**Conclusions:**

Our liquid biopsy assay provides robust detection of cCAFs in mPDAC and mGI patients. The measurement of both circulatory stromal parameters, cCAFs and HA, adds valuable clinical information as they are associated with an unfavorable outcome in PDAC. These results highlight that stromal characteristics unique to PDAC could be leveraged to fill the current gap in discovering predictive biomarkers.

**Supplementary Information:**

The online version contains supplementary material available at 10.1186/s13046-024-03262-x.

## Background

With a 5-year survival rate of approximately 13% across all stages, pancreatic ductal adenocarcinoma (PDAC) remains a highly lethal malignancy with a devastating prognosis [1]. Incidences continue to rise and by 2040, PDAC is expected to be the second leading cause of cancer-related deaths in the Western world [2]. Missing or non-specific symptoms, limitations in imaging for early-stage detection and lack of reliable biomarkers often lead to diagnosis at an advanced stage [3]. Long-term survival, although exceedingly low, is mainly driven by the proportion of resectable tumor stages, while only 15–20% of patients are eligible for surgery at time of diagnosis [3, 4]. In advanced or metastatic stages, a median overall survival (mOS) of only 8 to 11 months has been reported [5, 6]. Low response rates to standard of care chemotherapy are in part attributed to the dense fibrotic stroma, a hallmark of the PDAC histopathology, that forms the bulk of the PDAC tumor volume and appears to hinder effective drug delivery [7, 8]. The stroma-rich tumor microenvironment contains a plethora of cellular and non-cellular components, including a wide range of different extracellular matrix proteins (ECM), notably hyaluronan (HA) and collagen types I, III, and IV [9]. Cancer-associated fibroblasts (CAFs) represent the predominant cell population within the stroma not only depositing ECM but rather acting as key regulators of molecular carcinogenesis [10, 11]. With the identification of distinct CAF subtypes, the concept of intratumoral CAF heterogeneity emerged, providing a new paradigm for understanding CAF biology in PDAC [12]. In a dynamic interplay with cancer cells, the diverse CAF subsets exhibit a notable range of functional diversity [11]. In contrast to the extensive studies on CAFs in the primary tumor micromilieu, knowledge of CAFs, that enter the bloodstream, known as circulating CAFs (cCAFs), in PDAC is limited. Preclinical studies have shown that, cCAFs can migrate in the circulation either independently or in heterotypic clusters with circulating tumor cells (CTCs). These studies on breast, lung, and prostate cancer imply that these aggregates facilitate the formation of metastatic lesions, e.g., by providing a survival advantage against shear stress [13–15]. Over the years, liquid biopsy has emerged as a crucial non-invasive tool for extracting information from tumors that are difficult to access [16]. Several studies have shown an association between the presence of CTCs and a worse survival in PDAC patients, but often with rather low sensitivity [17–21]. The concept of cCAFs as a surrogate for tumor-associated stroma has become a promising area within liquid biopsy research [22–24] and, especially in context of the stroma-dense PDAC, cCAFs hold substantial promise as an innovative new biomarker. Due to the large morphologic heterogeneity and the lack of a specific fibroblast marker, standardized cCAF identification methods are not yet available [25].

The aim of our study was to establish and validate a liquid biopsy assay for the simultaneous detection of stromal analytes and CTCs in patients with PDAC and compare it with other gastrointestinal malignancies (GI) known to display different tumor-stroma ratios [26, 27]. In this exploratory study, we report on cCAF and HA levels and their potential adverse prognostic value in patients with PDAC. Moreover, we examined levels of these stromal analytes in relation to their expression within the corresponding tumor tissues. Given the limited data on the clinical significance of cCAFs in PDAC, we focus our research on advanced disease stages. Our findings extend the current knowledge of stromal analytes as potential predictive biomarkers in PDAC.

## Methods

### Cells and culture conditions

The primary human PDAC CAF cell lines LueCAF11 and LueCAF18 were generated by outgrowth method from human PDAC tissue and maintained in Dulbecco’s modified Eagle’s medium (DMEM, high glucose, GlutaMAX™, Gibco™) with 10% Fetal Bovine Serum (FBS, Capricorn scientific) and 1% Penicillin/Streptomycin (P/S, Gibco™) [28]. The MCF-7 cell line was obtained commercially from ATCC (ATCC, Manassas, VA, USA). To culture MCF-7 cells, DMEM medium (Gibco™) was supplemented with 10% FBS, 1% L-glutamine (Gibco™) and 1% P/S. All cell lines were cultured within a CO_2_-humidified incubator at 37 °C and underwent, prior to experiments, regular testing for *Mycoplasma* using the Venor GeM Mycoplasma Detection Kit (Minerva Biolabs).

### Spiking of healthy donor blood

Whole blood of HD was provided by the Department of Transfusion Medicine, University Medical Center Hamburg-Eppendorf (UKE) and used for the isolation of peripheral blood mononuclear cells (PBMCs). PBMCs were purified via Ficoll density gradient-based Leucosep™ enrichment (Greiner Bio One) and solved in Phosphate-Buffered Saline (PBS, Gibco™). Before spiking, LueCAF11 and LueCAF18 were labelled with CellTracker ™ Green CMFDA (Thermo Fisher Scientific) and MCF-7 with CellTracker™ Orange CMTMR (Thermo Fisher Scientific) according to the manufacturer's instructions. Subsequently, cells were spread on a Petri dish filled with PBS, hand-counted and collected under a light microscope. First, to determine the recovery rate of each enrichment method separately (*n* = 3), predetermined cell counts of manually picked 100 LueCAF11 or LueCAF18 cells were added to 1 × 10^7^ PBMCs for MACS® enrichment and to 7.5 mL EDTA blood for Parsortix™ analyses. To test the sequential enrichment assay (*n* = 4), a heterologous cell mixture was prepared by adding 100 MCF-7 with 100 LueCAF11 or 100 LueCAF18 cells to 1 × 10^7^ PBMCs blood each. The MCF-7 cell line was previously utilized to standardize a CTC enrichment protocol based on the Parsortix™ technology and serves as a surrogate for CTCs in our spike-in experiments [29].

### Study cohort and sample collection

For measurement of stromal analytes 7.5 mL EDTA blood samples were collected from patients treated at the UKE with localized PDAC prior to curative surgery (UICC I-III, *n* = 29), with metastatic PDAC (mPDAC, stage UICC IV, *n* = 27) and with metastatic GI (mGI, all UICC stage IV, total *n* = 23; metastatic colorectal carcinoma (mCRC, *n* = 11), metastatic gastric cancer and gastroesophageal junction adenocarcinoma (mGC, *n* = 8) and metastatic cholangiocellular carinoma (mCCA, *n* = 4)) at the time of initial diagnosis or disease progression. Samples from *n* = 22 patients in the mPDAC group and *n* = 23 samples in the mGI group were included in the cCAF analysis, with two sequential samples obtained from patients with mPDAC. For HA measurement blood samples from HD (*n* = 20), patients with localized PDAC (*n* = 29) and patients with mPDAC (UICC IV, *n* = 24 patients) were included. Follow-up samples from four mPDAC patients were also processed for the HA analysis (Supplementary Table 1). Corresponding tumor tissue sections from 14 patients with mPDAC were analyzed to compare biomarker levels in blood with the expression of αSMA, FAPα, and the HA extent in corresponding tumors using immunohistochemistry (IHC).

All blood samples were processed within two hours of collection. The blood sample collection for the cohort of metastatic patients began in November 2021 and for the cohort of localized PDAC patients in February 2020. Furthermore, data on clinical-pathological characteristics were gathered for each patient. Data were cut-off as of February 28, 2024, and missing endpoints were recorded by cross-checking data provided by the official registration authorities. Approval for clinical data collection and biobanking was granted by the Ethics Committee of Hamburg (Ethics Nr. PV5119 and PV3548). Written consent was obtained from all participants prior to blood donation.

### Plasma HA analysis

Plasma was isolated from EDTA blood using a 2-step centrifugation protocol (10 min at 300 g and 10 min at 1800 g) and stored at −80 °C until use. Levels of plasma HA were determined using the Quantikine® ELISA Hyaluronan Immunoassay (R&D Systems Inc., Minneapolis, USA) according to the manufacturer’s instructions.

### Spiked blood and patient sample processing for cCAF and CTC analysis

The spiked samples were further processed using two different enrichment methods: First, positive selection of CAFs from the PBMCs was performed with human Anti-Fibroblast MicroBeads (D7-FIB-conjugated, Miltenyi Biotec) using MS Columns (MACS® cell separation, Miltenyi Biotec) following the manufacturer’s instruction. As a comparative, marker-independent technique for processing the spiked EDTA blood, we employed the microfluidic Parsortix™ cell separation system (Angle plc, UK). This device allows size and deformability-based enrichment of blood cells with a diameter of > 6.5 μm, that has been successfully established in the field of CTC enrichment including analyses of PDAC patients [29–32]. Finally, we integrated both approaches in a sequential assay to enhance the output and capture non-D7-FIB-conjugated cells as well. After separation of the magnetically labeled cells, the flow-through containing the unlabeled cells was collected for further processing with Parsortix™. Subsequently, cells enriched with each single method and the combined approach were harvested into cytospin funnels, centrifuged onto a glass slide and processed immediately. After the samples from the spike experiments underwent ethanol fixation (99%, Sigma Aldrich, Steinheim, Germany), DAPI (Sigma Aldrich, Steinheim, Germany, 1:1000) was used for nuclear staining. Cells were enumerated under a fluorescence microscope, with CellTracker™ Green CMFDA/ CellTracker™ Orange CMTMR positive and DAPI-positive cells with intact morphology being included in the evaluation.

From whole blood of patient samples, the total cellular fraction was collected using Ficoll density gradient-based Leucosep™ enrichment. This fraction was subsequently processed using the above-mentioned sequential enrichment methods. Both the cells after magnetic separation (MACS® cell separation) and after enrichment using Parsortix™ were harvested in cytospin funnels, centrifuged onto glass slides, dried overnight, and stored at 80 °C prior to further processing. If the quantity of PBMC counts surpassed 1 × 10^7^ PBMCs, the samples were partitioned and subjected to further processing on an additional MS column to prevent exceeding the assay's limit for positive selection.

### Cell detection in patient samples

The composition of antibody panels for immunofluorescence staining of processed patient samples differed depending on the enrichment method employed. Slides were brought to room temperature and fixed with 4% PFA (Sigma Aldrich, Steinheim, Germany) for 10 min. After permeabilization with 0.1% Triton X (Sigma Aldrich, Steinheim, Germany) for 10 min samples were blocked for 30 min with 10% AB-serum/PBS (BioRad, Rüdigheim, Germany). For the staining of the enriched cells after MACS® cell separation unconjugated mouse anti-human αSMA (clone 1A4, ab7817, Abcam, 1:500) and rabbit anti-human FAPα (clone E1V9V, Cell Signaling, 1:50) antibodies were incubated over night at 4 °C. Rabbit anti-mouse (Alexa Fluor™ 488, Invitrogen, A-11059, 1:200) and goat anti-rabbit (Alexa Fluor™ 488, Invitrogen, A-11008, 1:200) were used as a secondary antibody and incubated for 60 min. Directly conjugated CD105 antibody (Sn6, PE, eBioscience™, Invitrogen, 1:200), CD45 antibody (clone H130, PerCP, BioLegend, 1:200) and DRAQ5™ for nuclear staining (Cell Signaling, 1:1000) were incubated for 60 min at room temperature. For the staining of the enriched cells after Parsortix™ cell separation the CD105 antibody was replaced by the pan-cytokeratin antibody (AE1/AE3, eFluor™ 570, Invitrogen, 1:200). Subsequently, cytospins were covered with Fluoromount-G™ Mounting Medium (Invitrogen), sealed with cover slips and examined using the fluorescence microscope. Under the condition of intact morphology, an αSMA^+^- and/or FAPα^+^-, AE1/AE3^−^-, CD45^−^-cell population was defined as cCAFs, whereas an αSMA^−^- and/or FAPα^−^-, AE1/AE3^+^-, CD45^−^-cell population was considered as tumor cells.

### Analysis of αSMA, FAPα and HABP expression in tissue samples

14 formalin-fixed paraffin-embedded (FFPE) tissue sections from PDAC tumors were analyzed for αSMA, FAPα and Hyaluronan-binding protein (HABP) using IHC. In brief, to quantify the two CAF marker expression (αSMA and FAPα), 4 µm tissue sections were deparaffinized, rehydrated, and subjected to heat-induced epitope retrieval (BioGenex, Antigen Retrieval Citra Plus Solution) and incubated with primary antibodies anti-αSMA (Abcam, mouse mAB ab7817, dilution 1:3000) and anti-FAPα (Cell Signaling rabbit mAB clone 66562S, dilution 1:100) in Antibody Diluent DAKO (Dako, Glostrup, Danmark) over night at 4° C. Dako REAL EnVision Detection System (K5007 Dako) was used to visualize the proteins. Hyaluronan deposition was detected in tissue sections using a biotinylated hyaluronic acid-binding protein (HABP) as previously described [33]. In brief, antigen retrieval was performed in a water bath at 60 °C over night in a 1:10 diluted DAKO retrieval solution (#S1699, Dako). Biotinylated hyaluronic acid binding protein (HABP, # 385,911 Calbiochem, Merck, Darmstadt, Germany) diluted 1:75 in Antibody Diluent (# B 1-31C, Medac, Wedel, Germany) was applied for 1 h at room temperature. The binding sites were detected using the ABC-AP-Kit (Vector Laboratories Inc., Burlingame, CA). The Permanent Red Kit (Dako) was used as a chromogen.

### Statistical analysis

The data were analyzed using IBM SPSS Statistics (Version 29) and GraphPad Prism (Version 9.4.1). Pearson's r (Pearson correlation coefficient) was employed to assess the correlation between cCAF and HA levels with the clinicopathologic data. Overall survival (OS) curves were plotted using the Kaplan–Meier method and compared by the Log-rank test. The OS was calculated as the period from sample collection until death or the last follow-up. The Cox proportional hazard regression model was employed for univariate and multivariate analysis to evaluate prognostic value of high cCAF and HA levels. Findings are reported as hazard ratios (HR) and 95% confidence intervals (CI). *P*-values of less than 0.05 with two-sided tests indicated significance.

## Results

### Establishment of a combined CAF and CTC liquid biopsy assay

Assuming that CAFs in circulation also exhibit a high degree of morphologic and biological variability similar to tissue, we assessed two distinct enrichment methods in comparative spike experiments. Therefore, we challenged the size and deformability-based cell separation system (Parsortix™) and secondly the marker-dependent enrichment technique (MACS® cell separation) with two morphological different primary PDAC CAF cells lines (Supplementary Fig. 1). Prior to cell capture, cell lines were transiently labeled to identify the spiked cells. The MACS®-based method resulted in mean recovery rates of 68% (standard deviation (SD) 5.0%, range: 63–73%) and 73% (SD 12.0%, range: 63–87%) for the primary human PDAC cell lines LueCAF11 and LueCAF18, respectively, whereas the Parsortix™ method yielded mean recovery rates of 57% (SD 9.6%, range: 50–68%) and 60% (SD 11.0%, range: 49–71%) (Table 1). Across both CAF cell lines, the MACS®-based approach demonstrated an average capture efficiency of 71% (SD 8.9%, range: 63–87%) as compared to 59% (SD 9.4%, range: 49–71%) using Parsortix™ (*P* = 0.047) (Supplementary Fig. 2). Notwithstanding the higher mean recovery rates of the MACS®-based enrichment method, we then integrated both techniques in a sequential liquid biopsy assay to extract potentially D7-FIB^−^-CAFs that evade capture through magnetic labeling (MACS®). The Parsortix™-System was employed for additional marker-independent processing of the flow-through after magnetic cell separation, which comprised magnetically unlabeled cells, thereby enabling the extraction of tumor cells. Subsequently, the combined enrichment approach was evaluated in a spike experiment including MCF-7 cells to complement the two CAF cell lines. Mean recovery rates of 62% (SD 12.0%, range: 49–75%) and 75% (SD 24.0%, range: 39–89%) were observed for the cell lines LueCAF11 and LueCAF18, respectively, using the sequential approach, with MCF-7 cells exhibiting a mean recovery rate of 60% (SD 17.0%, range: 27–76%) (Table 1).

**Table 1:** Mean CAF and CTC recovery rates

	**Enrichment method**						
	**MACS®**		**Parsortix™**		**Sequential assay**		
Cells	LueCAF11	LueCAF18	LueCAF11	LueCAF18	LueCAF11	LueCAF18	MCF-7
Mean Recovery [%]	68	73	57	60	62	75	60
SD [%]	5.0	12.0	9.6	11.0	12.0	24.0	17.0
Range [%]	63–73	63–87	50–68	49–71	49–75	39–89	27–76
N	3	3	3	3	4	4	8

Mean CAF and tumor cell recovery rates determined from spike experiments using either MACS® or Parsortix™ cell separation or the sequential approach. Percentage mean and range of recovery rates as well as the standard deviation (SD) are indicated. N: Number of technical replicates

### Characteristics of study population

The study included patients with localized (*n* = 29) and metastatic (*n* = 27) PDAC as well as other metastatic GI malignancies (*n* = 23). Corresponding clinicopathologic data are reported in Supplementary Table 1. Patients with localized PDAC provided samples prior to curative surgery, the majority (*n* = 19, 65.5%) of whom were diagnosed at UICC stage I-II. Samples in the metastatic cohorts were mainly drawn at the point of initial diagnosis (mPDAC group: *n* = 19/27, 70.4%; mGI group: *n* = 14/23, 60.9%), with a smaller proportion being obtained upon disease progression (Supplementary Table 1). Within our HA analysis we incorporated a cohort of 20 healthy blood donors, comprised of 9 women and 11 men, at a mean age of 63 years (SD 4.3 years, range: 56–70 years).

### Cellular stromal and tumor-derived components: analysis of CTCs, cCAFs and tumor tissue

**Fig. 1:**
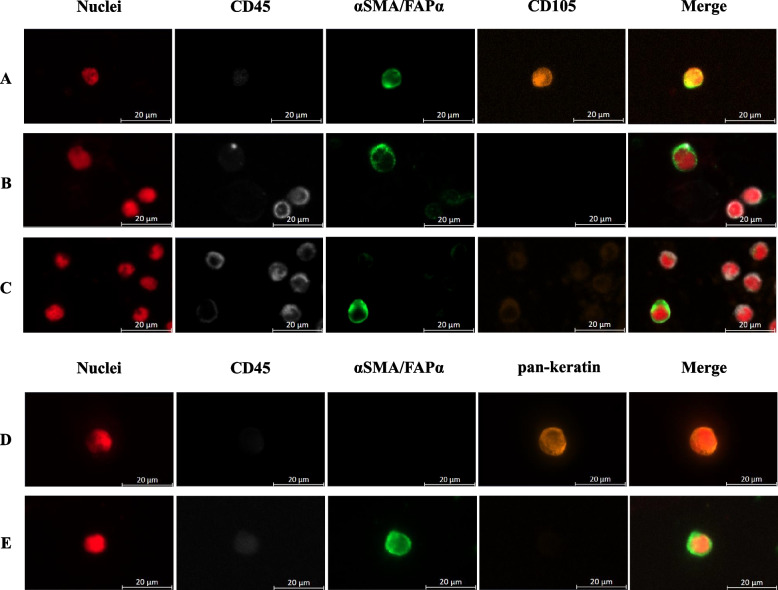
Example of fluorescence microscopy of circulating cancer-associated fibroblasts (cCAFs) and circulating tumor cells (CTCs). CD105 + cCAF (**A**) versus CD105- cCAFs (**B**, **C**), CTC (**D**), cCAF (**E**). Red: nuclei. White: CD45. Green: αSMAand/or FAPα, Orange: CD105 and AE1/AE3 (pan-keratin). The scale bar of 20 μm applies to all pictures

**Fig. 2:**
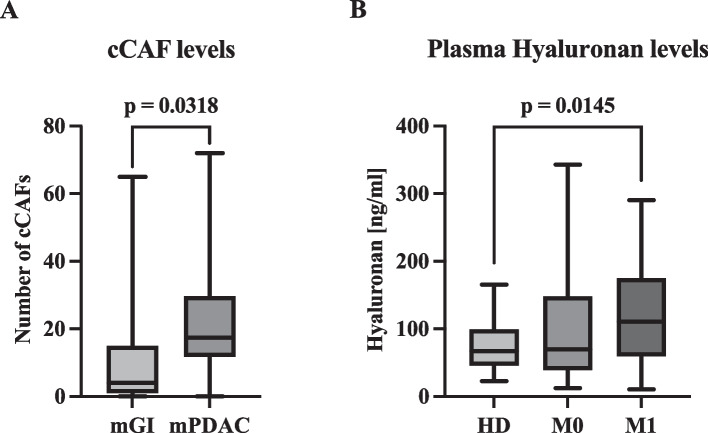
**A** Mean cCAF levels in patients with mPDAC (*n* = 22) compared to patients with mGI (*n* = 23). Abbr.: mPDAC: Metastatic pancreatic ductal adenocarcinoma, mGI: Metastatic gastrointestinal malignancies, cCAF: Circulating cancerassociated fibroblast. **B** Plasma Hyaluronan (HA) levels in healthy blood donors (*n* = 20, HD), localized (*n* = 29) and metastatic (*n* = 24) PDAC (Pancreatic ductal adenocarcinoma). Abbr.: M0 indicates localized and M1 metastatic PDAC

Our sequential liquid biopsy assay was employed to process samples from 22 patients with mPDAC and 23 patients with mGI. Subsequently, the enriched cells were identified by multiplex immunofluorescence, thereby defining cCAFs as an αSMA^+^- and/or FAPα^+^-, AE1/AE3^−^-, CD45^−^-cell population (Fig. 1). cCAFs (≥ 1 cCAFs/7.5 mL EDTA blood) were detected in 95.4% (*n* = 21/22) of patients with mPDAC and in 78.2% (*n* = 18/23) of patients with mGI (Table 2). In mPDAC patients, the average number of cCAFs per 7.5 mL EDTA blood (mean number 22.7; range: 0–72) showed a significantly higher value (*P* = 0.0318) compared to mGI patients (mean number 11.0; range: 0–65) (Fig. 2A, Table 2). As a potential discriminatory marker for two functionally divergent CAF subtypes, we analyzed the expression of CD105 within the cCAF population (Fig. 1A) [34]. In particular, the detection rate (≥ 1 CD105^+^ cCAF/7.5 mL EDTA blood) was comparable between patients with mPDAC (*n* = 4/22; 18.1%) and mGI (*n* = 4/23; 17.4%) (Table 2). Although our sequential liquid biopsy assay permitted the simultaneous detection of cCAFs and CTCs in a single sample, the detection rate and mean values of CTCs in both groups were low. However, CTCs (≥ 1CTC/7.5 mL EDTA blood) were identified in 22.7% of patients with mPDAC (*n* = 5/22; mean 0.45; range: 0–5) and in 30.4% of patients with mGI (*n* = 7/23; mean: 0.28; range: 0–1) (Table 2). To determine whether cCAF levels correlate with corresponding tumor tissue expression, we assessed the expression levels of the CAF markers FAPα as well as αSMA using IHC (Supplementary Fig. 3). High expression levels of FAPα and αSMA were observed in most PDAC tissues. Remarkably, of the 14 tissue sections analyzed, ten demonstrated high expression of at least one of the two cCAF markers, and no tissue section was negative for both markers, indicating their potential use as universal marker within the PDAC tissue (Supplementary Table 2). Of note, no correlation was observed between the tissue expression levels of FAPα and αSMA and blood levels of cCAF. This finding suggests that the release of this stromal analyte may be independent of its tissue abundance.

**Table 2:** Levels of detected cCAFs and CTCs in the study groups

	**mPDAC**	**mGI**
N	22	23
cCAFs		
cCAF positive [%]	21 [95.4]	18 [78.2]
Mean N [range]	22.7 [0–72]	11.0 [0–65]
Groups by cut-off [< 15/ ≥ 15]	9/13	17/6
CD105^+^ cCAF [%]	4 [18.1]	4 [17.4]
Mean N [range]	0.36 [0–3]	0.39 [0–6]
CTCs		
CTC positive [%]	5 [22.7]	7 [30.4]
Mean N [range]	0.45 [0–5]	0.28 [0–1]

Levels of detected circulating cancer-associated fibroblasts (cCAFs) and circulating tumor cells (CTCs) in the study groups. *Abbreviations*: *mPDAC* Metastatic pancreatic ductal adenocarcinoma, *mGI* Metastatic gastrointestinal malignancies, *SD* Standard deviation, *N* Number

### HA levels in healthy blood donors and patients with localized and metastatic PDAC

Furthermore, as an additional stromal analyte, we evaluated plasma HA levels in healthy blood donors as well as patients with localized and metastatic PDAC (Table 3). Mean HA levels in patients with mPDAC (123.0 ng/mL; range: 10.3–290.7 ng/mL) were significantly (*P* = 0.015) higher compared to HD (74.5 ng/mL; range: 22.8–165.5 ng/mL) (Fig. 2B). In localized PDAC patients, mean plasma HA levels showed a non-significant higher trend (105.1 ng/mL; range: 12.4–343.1 ng/mL; *P* = 0.157) than in the HD group. The median plasma HA level was 110.8 ng/mL (IQR: 59.3–175.3 ng/mL) in metastatic and 69.4 ng/mL (IQR: 38.9–148.2 ng/mL) in localized PDAC patients.

**Table 3:** Plasma HA levels in the study groups

	**HD**	**PDAC**	
		**localized**	**metastatic**
N	20	29	24
Median [ng/ml, IQR]	67.0 [45.6–99.2]	69.4 [38.9–148.2]	110.8 [59.3–175.3]
Mean [ng/ml, range]	74.5 [22.8–165.5]	105.1 [12.4–343.1]	123.0* [10.3–290.7]
SD	34.7	90.7	78.9

Plasma Hyaluronan (HA) levels in healthy blood donors (HD), localized and metastatic pancreatic ductal adenocarcinoma (mPDAC)

*Abbreviations*: *N* Number, *IQR* Interquartile range, *SD* Standard deviation. *P = 0.015 compared to healthy controls

Subsequently, to explore a potential correlation between plasma HA levels and HA expression in corresponding tumor tissues, we assessed the expression levels of HABP as a surrogate marker for HA extent in the tissue slides of 14 mPDAC patients (Supplementary Fig. 3) [33]. Three cases with HA values exceeding the third quartile (> 148.2 ng/mL) exhibited high levels of HABP tissue expression. By employing the median HA level (> 69.4 ng/mL) as a threshold, five cases were associated with pronounced tissue HABP expression, indicating a rather correlation between circulating HA and tissue expression (Supplementary Table 2). Given the scarce availability of matching samples, statistical analysis of this correlation was not performed.

### Clinical Value and prognostic relevance of cCAFs, CTCs and HA levels

**Fig. 3:**
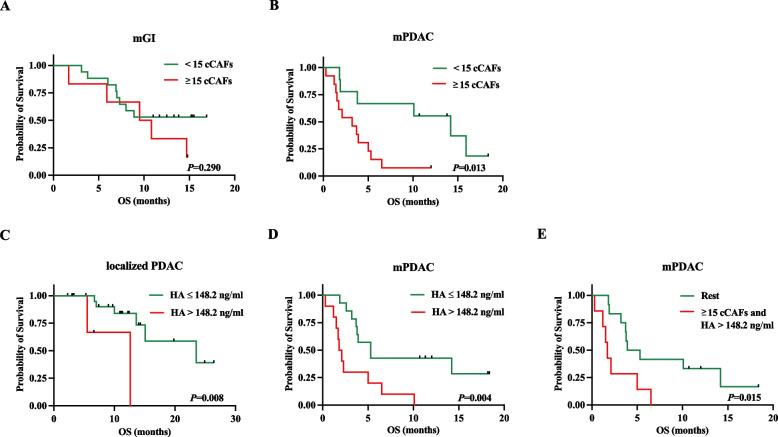
Kaplan Meier curves for overall survival stratified by cCAF and HA plasma levels. Patients at risk: **A** *n* = 23 (17 vs. 6); **B** *n* = 22 (9 vs. 13); (**C**) *n* = 29 (22 vs. 7); (**D**) *n* = 24 (14 vs. 10), and (**E**) *n* = 19 (12 vs. 7). Abbr.: cCAF: Circulating cancer-associated fibroblast, HA: Hyaluronan, mGI: Metastatic gastrointestinal malignancies, mPDAC: Metastatic pancreatic ductal adenocarcinoma

Given that in a majority of patients with mPDAC cCAFs were present, we investigated the impact of the cCAF levels on the mOS in our study groups (Fig. 3, Supplementary Table 3). A significant difference was observed in the mOS (*P* = 0.013 by log-rank test) of patients with mPDAC harboring ≥ 15 cCAFs per 7.5 mL of blood, with a median mOS of 3.2 months (95% confidence interval (CI) 0.801–5.855), in comparison to mPDAC patients with < 15 cCAFs, showing a median mOS of 14.2 months (95% CI 6.055–22.332) (Fig. 3B, Supplementary Table 3). In contrast levels of cCAFs ≥ 15 had no significant impact on mOS in mGI patients (9.5 months (95% CI 3.698–15.292); *P* = 0.290 by log-rank test) (Fig. 3A, Supplementary Table 3). Also, CTC levels were not associated with survival in either mPDAC or mGI patients (data not shown).

Next, we also hypothesized a relationship between HA levels and overall survival in our study group (Fig. 3, Supplementary Table 3). Therefore, we separated the cohort of localized PDAC patients along the third quartile (Q3) of HA levels (Q3 = 148.2 ng/mL). Patients with localized PDAC in the top quartile showed a significantly shorter mOS (12.6 months (95% CI not defined)) compared to patients with HA levels ≤ 148.2 ng/mL (23.5 months (95% CI 7.304–39.678), *P* = 0.008 by log rank test) (Fig. 3C, Supplementary Table 3). Subsequently, the cut-off of 148.2 ng/mL (corresponding to the third quartile of HA levels in the group with localized PDAC) was applied to the cohort of patients with mPDAC. Patients with mPDAC and HA levels > 148.2 ng/mL displayed a significant shorter mOS than patients with HA levels ≤ 148.2 ng/mL (mOS 1.774 months (95% CI 1.163–2.385) vs. 5.290 months (95% CI 2.786–7.794); *P* = 0.004 by log rank test) (Fig. 3D, Supplementary Table 3). As a combined approach, we then investigated the influence of both biomarkers on the mOS of patients with PDAC. In cases where both ≥ 15 cCAFs and HA levels > 148.2 ng/mL were observed, mOS of patients with mPDAC was significantly decreased (mOS 1.708 months (95% CI 1.118–2.299); *P* = 0.015) compared to patients below the double cut-off criteria (mOS 3.910 months (95% CI 1.344–6.475)) (Fig. 3E).

Univariate analysis showed that absolute number of cCAFs per 7.5 mL of blood (HR 1.057, 95% CI 1.020–1.095; *P* = 0.002) and HA levels > 148.2 ng/mL (HR 3.651, 95% CI 1.419–9.392; *P* = 0.007) were associated with worse prognosis in patients with mPDAC (Table 4). In our localized PDAC cohort patients with HA levels in the top quartil (> 148.2 ng/mL) (HR 8.175, 95% CI 1.306–51.171; *P* = 0.025) exhibited a diminished likelihood of survival (Supplementary Table 4). Finally, to verify the independent role of cCAF as a predictive biomarker for patients with mPDAC, we conducted multivariate analysis to eliminate potential confounding variables (Table 4). Multivariate analysis confirmed the absolute number of cCAFs as an independent predictor of mOS in patients with mPDAC (HR 1.047, 95% CI 1.001–1.096; *P* = 0.046). However, in our multivariate analysis high plasma HA levels (> 148.2 ng/mL) were not associated with a shorter mOS either in the cohort of patients with mPDAC (HR 1.656, 95% CI 0.526–5.214; *P* = 0.389) or with localized PDAC (HR 3.782, 95% CI 0.554–25.822; *P* = 0.175) (Table 4 and Supplementary Table 4).

**Table 4:** Univariate and multivariate Cox analyses for overall survival of the mPDAC cohort according to clinical characteristics and cCAF and plasma HA levels

		**Univariate analysis**		**Multivariate analysis**	
**Variable**	**N**	**HR (95% CI)**	***P*-value**	**HR (95% CI)**	***P*-value**
Total cCAFs	22	1.057 [1.020–1.095]	**0.002**	1.047 [1.001–1.096]	**0.046**
HA > 148.2 ng/ml (vs. ≤ 148.2 ng/ml)	10/14	3.651 [1.419–9.392]	**0.007**	1.656 [0.526–5.214]	0.389
CA19-9 ≥ 500 kU/l (vs. < 500 kU/l)	17/10	3.601 [1.178–11.011]	**0.025**	3.657 [0.680–19.666]	0.131
Age ≥ 75 years (vs. < 75 years)	6/21	2.989 [1.108–8.063]	**0.031**		
Sex (Female vs. male)	13/14	0.719 [0.303–1.706]	0.455		
ECOG ≥ 2 (vs. 0–1)	6/21	7.584 [2.449–23.485]	** < 0.001**		
Liver metastasis (Yes vs. no)	15/12	1.505 [0.609–3.718]	0.376		
Peritoneum metastasis (Yes vs. no)	10/17	2.080 [0.839–5.154]	0.114		
Prior lines of chemotherapy (0 vs. ≥ 1)	8/19	0.899 [0.347–2.333]	0.828		

Univariate and multivariate Cox analyses for overall survival of the mPDAC cohort according to clinical characteristics and cCAF and plasma HA levels

*Abbreviations*: *cCAF* Circulating cancer-associated fibroblast, *HA* Hyaluronan, *CI* Confidence interval, *mGI* Metastatic gastrointestinal malignancies, *mPDAC* Metastatic pancreatic ductal adenocarcinoma, *ECOG* Eastern Cooperative Oncology Group, *CA19-9* Carbohydrate antigen 19–9

It is noteworthy that cCAF numbers did not correlate with HA levels, nor did both biomarkers correlate with levels of the tumor marker CA19-9 (data not shown). In four patients with mPDAC, we obtained sequential samples at disease progression (Supplementary Table 5). An increase in HA values was observed in all four patients. The number of cCAFs and CTCs demonstrated a heterogenous pattern during disease progression. In one patient, cCAFs exhibited a stable count, while in two patients, CTCs demonstrated a clear trend towards an increase at the time of clinical progression.

## Discussion

The unmet need for clinically relevant predictive biomarkers in PDAC contributes notably to the persistent poor prognosis of these patients. Moreover, as stroma-targeted therapeutic approaches advance, the detection of also novel stromal related biomarkers could be requisite to enable stratification of treatments [5]. Currently, CA19-9 is an important biomarker employed in clinical practice, yet it exhibits notable diagnostic shortcomings. About 10% of patients lack the Lewis antigen resulting in false-negative interpretations of test results [35]. In addition, CA19-9 levels can be affected by conditions, such as pancreatitis or cholestasis, and even be elevated in the absence of malignancy or pancreatobiliary diseases [36–38].

Here we focused on tumor stroma-derived cellular and non-cellular signatures as potential targets for liquid biopsy approaches in PDAC. We postulate that via blood-based approaches characteristics of the tumor stroma can be exploited to identify potential novel and clinically easily usable biomarkers as an important tool for further treatment stratification. In this study, we established a non-invasive liquid biopsy assay to detect cCAFs in patients with mPDAC and other gastrointestinal malignancies. Our analysis demonstrated a strong association between increased levels of cCAFs as well as plasma levels of HA and markedly reduced overall survival in PDAC patients.

Although, the significance of CAFs, the most abundant cellular component in the PDAC stroma, is becoming more widely acknowledged, yet there exists a notable gap in our understanding of cCAFs and their relevance in clinical contexts. Given the extensive morphological variations and the absence of a distinct fibroblast marker, the task of utilizing cCAFs for liquid biopsy is inherently complex [24]. Therefore, the comparability of the few previous studies and their predictive value on the cCAFs frequency have been hampered by employing different surface markers and enrichment methods [22, 23, 39–41]. We have therefore successfully developed and validated a liquid biopsy assay that provides reliable detection of cCAFs in patients with mPDAC and other mGIs. First, for our spike experiments, we employed two morphological distinct primary PDAC CAF cell lines. Average detection rates across both CAF cell lines reached 71% with the MACS® cell separation system and 59% with the Parsortix™ technology indicating a lower capture efficiency for the size-based selection. Of note, the antigen D7-FIB used in the MACS® separation, is described as a general mesenchymal stem cell marker [25, 42]. The sole use of the aforementioned method carries thus a risk of omitting non-D7-FIB-conjugated cCAFs. To exclude this, we finally combined both approaches in a sequential liquid biopsy assay in order to cover the widest possible range of CAF heterogeneity. Furthermore, this allowed us to combine the assay with simultaneous detection of CTCs.

For cell identification via multiplex immunofluorescence, we selected the combination of surrogate markers αSMA and FAPα to capture the majority of cCAFs. These markers have been widely accepted and commonly utilized in previous research [22, 23, 25, 43]. Furthermore, to avoid inaccurately overestimating cCAF counts we applied double negativity for AE1/AE3 and CD45 in the precise definition of cCAFs [25]. This approach is crucial as certain subgroups of leukocytes are known to be partially FAPα^+^ and may represent tumor-associated macrophages [44]. Although vimentin was utilized in another study, we decided against its use, given the difficulties in distinguishing from CTCs undergoing epithelial-mesenchymal transition (EMT) or other cells of mesenchymal origin [45, 46].

In our work, we could successfully detect cCAFs in the blood of patients with mPDAC and mGI and demonstrated that cCAFs occur in over 95% of patients with mPDAC and 78% in patients with mGI. Most striking was the significant difference in mean cCAF numbers in mPDAC (22.7/7.5 mL EDTA blood) compared to mGI patients (11.0/7.5 mL EDTA blood). These results suggest that the presence of cellular stromal analytes is a potential feature of patients with mPDAC reflecting the high stromal abundance of the primary tumor site.

Data on cCAFs in patients with PDAC are scarce and, to our knowledge, only a previous study by Ortiz-Otero et al. investigated cCAFs in patients with PDAC [23]. In a heterogeneous cohort, including five patients with PDAC, notably high cCAF values were reported, with 98% of the samples surpassing 17 CAF/mL blood, whereas our data referred to 7.5 ml of EDTA blood [23]. In other studies, mainly on breast and prostate cancer patients, highly variable cCAF detection rates have been reported ranging between 3–100% [22, 23, 39, 40, 43]. The different detection rates might be explained by both the different detection methods employed and various tumor entities with different stromal involvement. However, the presence of cCAFs was demonstrated to be tumor specific, as no cCAFs or CAF-associated proteins were observed in healthy subjects according to previous studies [22, 23, 39, 41, 43, 47].

Numerous proteins linked to functionally divergent CAF subtypes in PDAC tissues have been elucidated in prior studies [12, 48, 49]. Here we analyzed the expression of CD105 in cCAFs, which has been identified as a potential discriminatory marker for a functionally specific CAF subtype with protumorigenic effects [34]. In our study, below 20% of the cCAFs were CD105 positive and did not have a significant impact on survival (data not shown) indicating that CD105 is not an optimal stratification marker for cCAFs. However, our findings indicate that cCAFs are frequently present in the bloodstream of mPDAC patients and therefore lay the groundwork for future studies exploring whether the heterogeneity of intratumoral CAFs is mirrored in cCAFs. Building upon this, it is conceivable that cCAFs may also exhibit functional variability and potentially hold different prognostic implications. Thus, subsequent investigations on cCAFs should incorporate subset discrimination and the identification of the cellular origins of αSMA- and FAPα-positive cells, for instance, by utilizing markers such as PDGFRα, Meflin, CD74 and IL-6 [12, 48, 49].

In our multi-marker approach, CTCs were detected in 22.7% of patients with mPDAC and in 30.4% of patients with mGI, a rate that is lower compared to findings from single-marker studies on CTCs [50]. As the average recovery rates of tumor cells (mean 60%) in our spike experiments were comparable to values reported in the literature, this result was unanticipated [29]. Obviously, spike experiments cannot fully mimic patient samples. However, our study extends beyond an exclusive focus on CTCs by placing significant emphasis on HA and cCAFs detection as novel combinatorial markers in PDAC, exploiting entity-specific stromal characteristics. Therefore, we implemented cCAF detection employing a combination of marker-dependent and -independent enrichment methods, to account for the large heterogeneity of PDAC CAFs. Nonetheless, a one-tube approach prioritizing accurate cCAF and HA detection may compromise CTC identification and is limited to cover the diverse range of stromal and tumor-derived analytes. To our knowledge only few studies have explored the simultaneous capture of CTCs and stromal cells in patients [22, 23, 39]. Two studies on breast cancer presented discrepant results, with CTCs present in 28% and 97% and cCAFs in 3% and 88% (*n* = 34) of the metastatic patients [22]. In a mixed study population, high levels of both cCAFs (98%) and CTCs (97%) were reported with no significant correlation between CTC levels and cancer prognosis [23]. These studies highlight that the comparability of studies is constrained by the application of varying detection techniques.

As an important clinical finding, our study provides evidence that high levels of cCAFs (cCAFs ≥ 15/7.5 mL EDTA blood) are associated with a significantly shorter mOS in mPDAC. To our knowledge, this study is the first to demonstrate the negative impact of high cCAF levels on mOS in patients with mPDAC. Univariate and multivariate analyses supported the finding that higher cCAF levels in patients with mPDAC is indicative of a higher likelihood of reduced mOS, whereas this association was not found for patients with other GI malignancies.

In addition, as another stroma-derived biomarker, we assessed HA levels in patients with metastatic and localized PDAC and compared them to those in healthy controls. Consistent with previous reports, mean HA plasma levels in our study were significantly higher in mPDAC patients compared to healthy subjects [51]. Notably, high HA levels were associated with significantly shorter mOS in both patients with localized and metastatic PDAC. Hence, this study supports high levels of HA as a negative predictive biomarker in PDAC. HA levels and cCAF levels did not appear to correlate with each other, indicating potentially independent biomarkers. Yet, high cCAF levels combined with high HA levels were predictive of a significantly poorer outcome in terms of reduced mOS in mPDAC.

In this pilot study, blood concentrations of cCAFs did not reflect tissue levels of CAFs. Moreover, while high expression levels of CAF markers suggest a universal presence of stromal cells within the tumor tissue, high cCAF levels in circulation may indicate a rather aggressive tumor phenotype. Although the small sample size did not allow for a statistical analysis of the correlation, several patient cases pointed to an association between HA values and HABP expression in tissue. Nonetheless, the mechanisms underlying the release of stromal analytes into circulation are still not understood. This points to the critical need for further exploration of these biomarkers and their correlations with tissues from both primary and metastatic sites in upcoming studies.

Clearly, the study presented is limited by the small number of patients included and thus, our study serves as an initial step in establishing cCAFs as a biomarker for PDAC. Still this study represents the largest reported cohort of mPDAC patients to date, encompassing a multimarker analyses approach. This proof-of-concept research provide a basis for further investigation into the prognostic relevance of cCAFs, not only in advanced stages but also in localized PDAC, particularly within the perioperative context. Additionally, future research should incorporate longitudinal assessments of stromal analytes with appropriate follow up to clarify their predictive capacity for treatment stratification and therapeutic response.

## Conclusion

In summary, we successfully developed a non-invasive liquid biopsy assay to detect cCAFs in patients with mPDAC and other GI malignancies. In our exploratory study, high cCAF and HA levels were both associated with significantly worse mOS in mPDAC. At present, the lack of predictive biomarkers for PDAC is a major challenge. It is therefore conceivable that PDAC-specific stromal characteristics could be exploited in the ongoing search for predictive biomarkers. Our data suggest that stromal analytes, in particular cCAFs and HA, may serve as adverse prognostic biomarkers in PDAC.

## Supplementary Information

Supplementary Material 1.

Supplementary Material 2.

## Data Availability

The data supporting the results of this study are available upon reasonable request from the corresponding authors.

## Abbreviations

αSMA: α-Smooth muscle actin
CAFs: Cancer-associated fibroblasts
CA19-9: Carbohydrate antigen 19–9
CCA: Cholangiocellular carcinoma
cCAFs: Circulating cancer-associated fibroblasts
CI: Confidence interval
CRC: Colorectal carcinoma
CTCs: Circulating tumor cells
DMEM: Dulbecco’s modified Eagle’s medium
ECM: Extracellular matrix proteins
ECOG: Eastern cooperative oncology group
EMT: Epithelial-mesenchymal transition
FAPα: Fibroblast activation protein-α
FBS: Fetal bovine serum
FFPE: Formalin-fixed paraffin-embedded
FUP: Follow up
GC: Gastric cancer
GI: Gastrointestinal malignancies
HA: Hyaluronan
HABP: Hyaluronan-binding protein
HD: Healthy blood donors
HR: Hazard ratios
IHC: Immunohistochemistry
IQR: Interquartile range
mCCA: Metastatic cholangiocellular carcinoma
mCRC: Metastatic colorectal carcinoma
mGC: Metastatic gastric cancer
mGI: Metastatic gastrointestinal malignancies
mOS: Median Overall survival
mPDAC: Metastatic pancreatic ductal adenocarcinoma
PBMC: Peripheral blood mononuclear cells
PBS: Phosphate-Buffered Saline
PDAC: Pancreatic ductal adenocarcinoma
P/S: Penicillin/Streptomycin
SD: Standard deviation
UICC: Union for International Cancer Control
UKE: University Medical Center Hamburg-Eppendorf
