# Supplementary material for: Identification and characterization of tumor and stromal derived liquid biopsy analytes in pancreatic ductal adenocarcinoma
Source: J Exp Clin Cancer Res. 2025 Jan 16;44:14. doi: 10.1186/s13046-024-03262-x (PMC11737273; doi:10.1186/s13046-024-03262-x)
Supplement: Supplementary file 1 — Supplementary Material 1. [file 13046_2024_3262_MOESM1_ESM.pptx]

## Slide 1
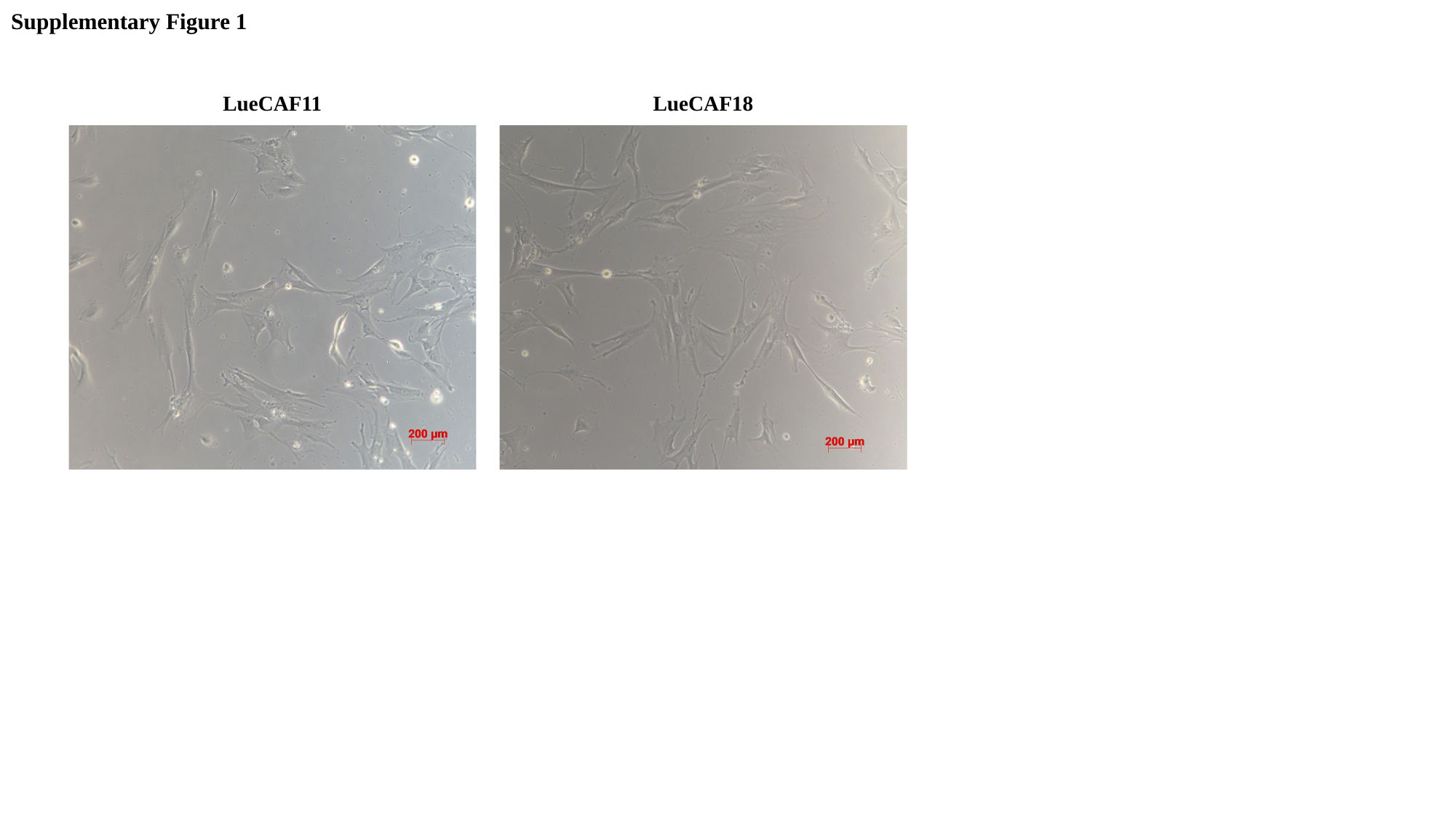

Supplementary Figure 1
LueCAF11
LueCAF18

## Slide 2
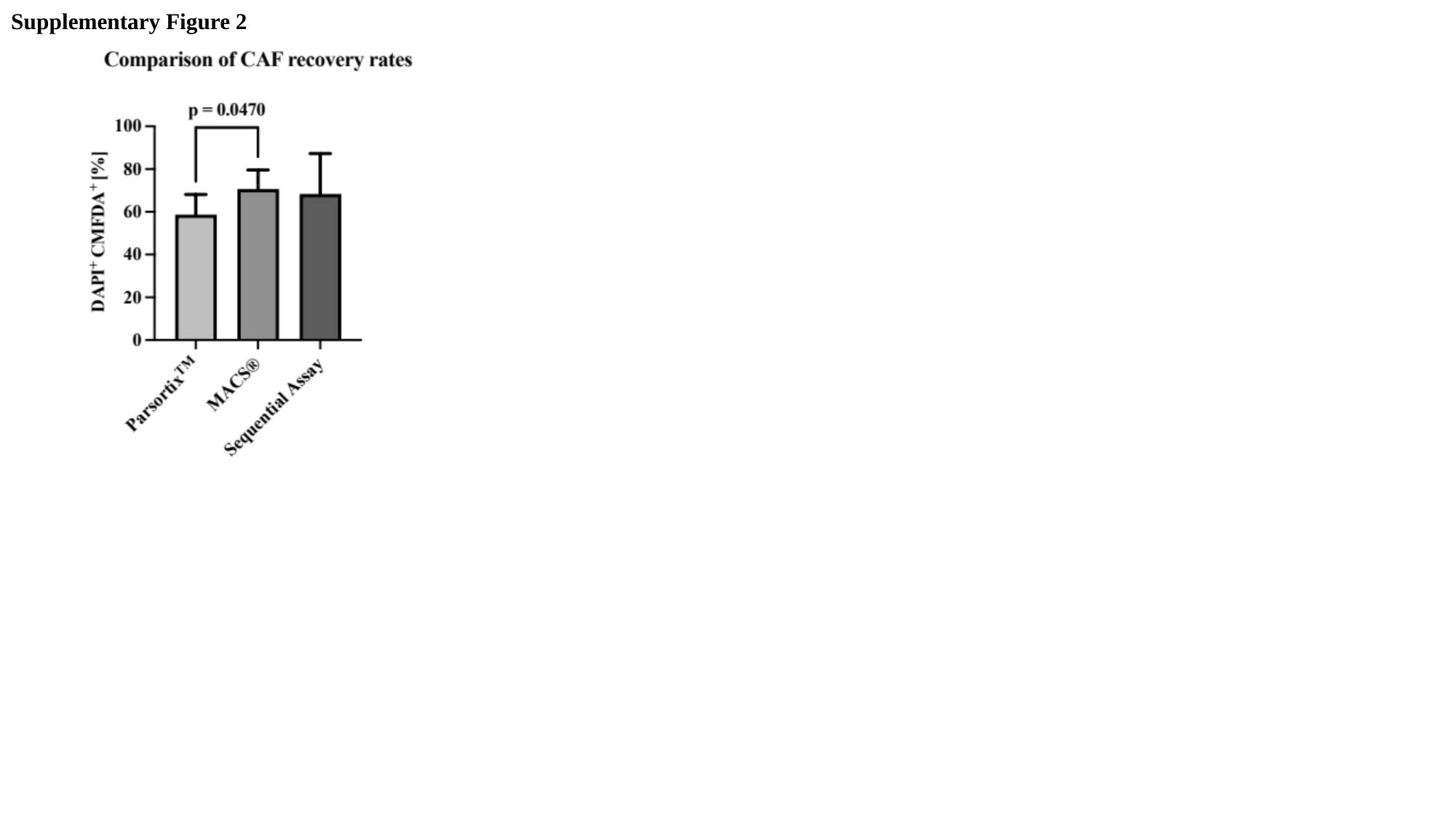

Supplementary Figure 2

## Slide 3
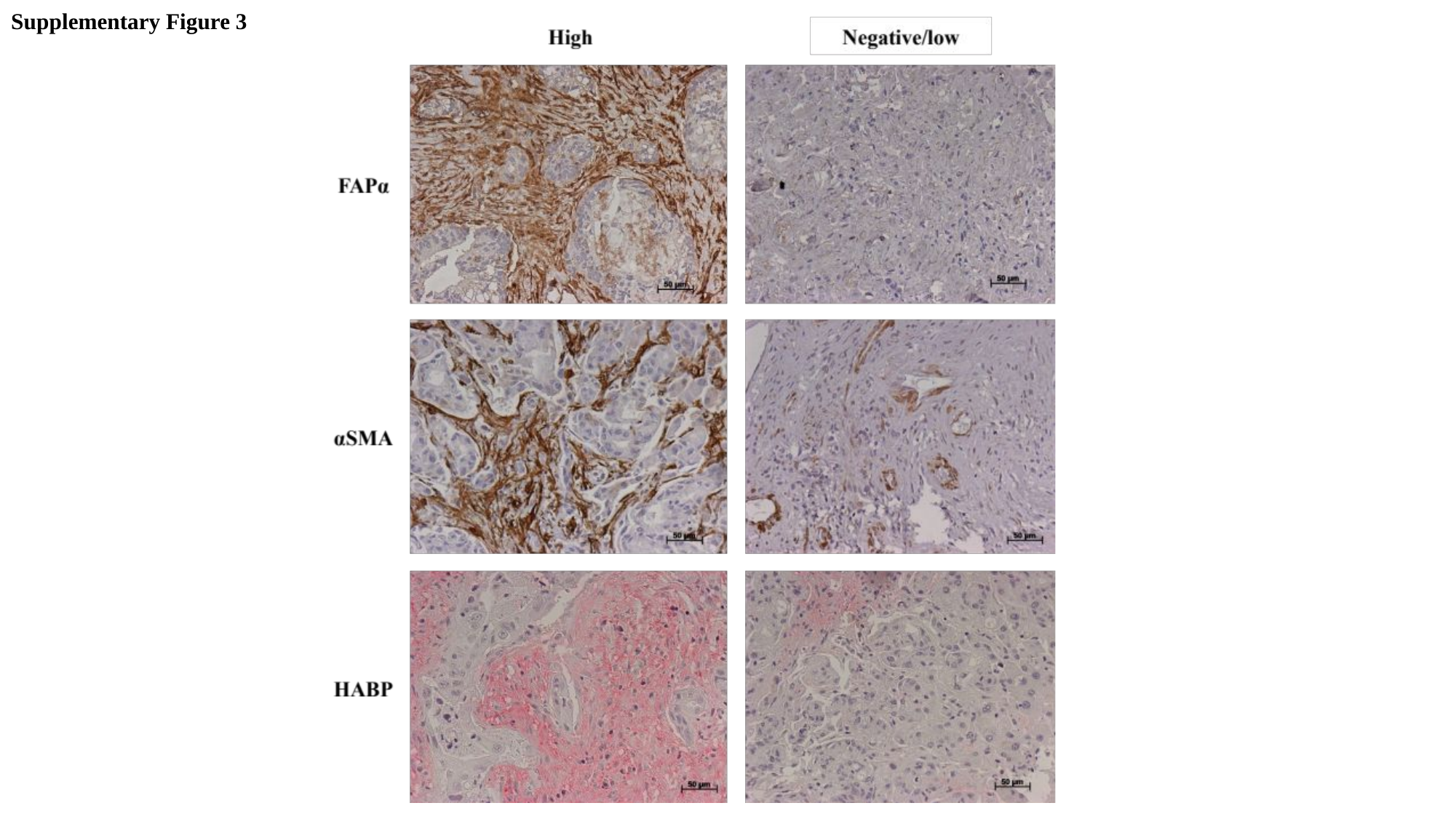

Supplementary Figure 3
