# Supplementary material for: Identification and characterization of tumor and stromal derived liquid biopsy analytes in pancreatic ductal adenocarcinoma
Source: J Exp Clin Cancer Res. 2025 Jan 16;44:14. doi: 10.1186/s13046-024-03262-x (PMC11737273; doi:10.1186/s13046-024-03262-x)
Supplement: Supplementary file 2 — Supplementary Material 2. [file 13046_2024_3262_MOESM2_ESM.docx]

**Supplementary Table 1:** *Characteristics of the study population*

|  | **localized PDAC** | | **mPDAC** | | **mGI** | |
| --- | --- | --- | --- | --- | --- | --- |
| **Characteristic** | **Number** | **[%]** | **Number** | **[%]** | **Number** | **[%]** |
| Patients | 29 | 100 | 27 | 100 | 23 | 100 |
| Sex [female/male] | 11/18 | 62.1/37.9 | 13/14 | 51.9/48.1 | 14/9 | 60.9/39.1 |
| Age [years, median and range] | 73 [46-86] | | 67 [31-86] | | 62 [27-83] | |
| Tumor entity |  |  |  |  |  |  |
| PDAC | 29 | 100 | 27 | 100 |  |  |
| CRC |  |  |  |  | 11 | 47.8 |
| GC |  |  |  |  | 8 | 34.8 |
| CCA |  |  |  |  | 4 | 17.4 |
| UICC stage |  |  |  |  |  |  |
| I-II | 19 | 65.5 |  |  |  |  |
| III | 10 | 34.5 |  |  |  |  |
| IV |  |  | 27 | 100 | 23 | 100 |
| ECOG |  |  |  |  |  |  |
| 0 | 20 | 69.0 | 10 | 37.0 | 6 | 26.1 |
| 1 | 6 | 20.7 | 11 | 40.7 | 15 | 65.2 |
| 2 or more | 3 | 10.3 | 6 | 22.2 | 2 | 8.7 |
| Metastatic sites |  |  |  |  |  |  |
| Hepatic |  |  | 15 | 55.6 | 18 | 78.3 |
| Pulmonary |  |  | 7 | 25.9 | 6 | 26.1 |
| Peritoneum |  |  | 10 | 37.0 | 7 | 30.4 |
| Other |  |  | 3 | 11.1 | 2 | 8.7 |
| Number of metastatic sites |  |  |  |  |  |  |
| 1/>1 |  |  | 18/9 | 66.7/33.3 | 10/13 | 43.5/56.5 |
| CA19-9 ≥ 500 [kU/l] | 6 | 20.7 | 17 | 63.0 | 4 | 17.4 |
| Sample collection |  |  |  |  |  |  |
| Diagnosis/Disease progression | 29/0 | 100/0 | 19/8 | 70.4/ 29.6 | 14/9 | 60.9/39.1 |
| Clavien-Dindo [0-2/3-4] | 16/13 | 55.2/44.8 |  |  |  |  |
| R status [R0/R1] | 21/8 | 72.4/27.6 |  |  |  |  |
| Systemic therapy |  |  |  |  |  |  |
| Adjuvant (yes/no/unkown) | 20/5/4 | 69.0/17.2/13.8 |  |  |  |  |
| Prior lines (0/1 or more) |  |  | 19/8 | 70.4/ 29.6 | 14/9 | 60.9/39.1 |
| Stromal analyte |  |  |  |  |  |  |
| cCAF: patients [sequential samples] |  |  | 22 [2] | | 23 [0] | |
| HA: patients [sequential samples] | 29 [0] | | 24 [4] | |  |  |
| OS [months, median and 95% CI] | 23.491 [12.205-34.777] | | 3.91 [1.792-6.027] | | 10.776 [3.614-17.859] | |
| FUP [months, median and 95% CI] | 11.302 [9.293-13.312] | | 13.799 [8.001-19.597] | | 14.818 [12.575-17.060] | |

**Supplementary Table 1:** Clinical characteristics of the study population. Abbr.: mPDAC: Metastatic pancreatic ductal adenocarcinoma, mGI: Metastatic gastrointestinal malignancies, CRC: Colorectal carcinoma, GC: Gastric cancer and gastroesophageal junction adenocarcinoma, CCA: Cholangiocellular carcinoma, UICC: Union for International Cancer Control, ECOG: Eastern Cooperative Oncology Group, CA19-9: Carbohydrate antigen 19-9, R: Residual tumor, cCAF: Circulating cancer-associated fibroblast, HA: Hyaluronan, OS: Overall survival, CI: Confidence interval, FUP: Follow up.

**Supplementary Table 2:** *Comparison of biomarker levels in blood and tissue expression of αSMA, FAPα and HABP*

|  | Biomarker levels | |  | Tissue expression | | |
| --- | --- | --- | --- | --- | --- | --- |
| Sample ID | cCAFs | HA [ng/ml] |  | αSMA | FAPα | HABP |
| 12768 | 16 | 179.44 |  | high | high | high |
| 12802 | 11 | 82.59 |  | high | neg/low | high |
| 12880 | n.d. | 122.63 |  | moderate | neg/low | high |
| 12913 | 38 | 65.85 |  | neg/low | moderate | high |
| 12938 | 72 | 288.26 |  | high | high | high |
| 12962 | 28 | 156.72 |  | high | neg/low | high |
| 12440 | n.d. | 10.34 |  | moderate | high | moderate |
| 12967 | 14 | 59.34 |  | moderate | neg/low | moderate |
| 13293 | n.d. | 110.38 |  | moderate | high | moderate |
| 13351 | 38 | 61.38 |  | neg/low | high | moderate |
| 13608 | n.d. | 32.8 |  | moderate | high | moderate |
| 12884 | 9 | n.d. |  | neg/low | neg/low | neg/low |
| 13317 | 66 | n.d. |  | moderate | high | neg/low |
| 13523 | 18 | 111.29 |  | high | moderate | neg/low |

Supplementary Table 2: Levels of circulating cancer-associated fibroblasts (cCAFs) and hyaluronan (HA) compared to tissue expression of αSMA, FAPα and hyaluronan binding protein (HABP) in patients (N=14) with metastatic pancreatic ductal adenocarcinoma. Abbr.: n.d.: not determined, neg: negative.

**Supplementary Table 3:** *Overall survival of the study groups stratified by cCAF and HA plasma levels*

|  | **Variable** | **N** | **Median Overall Survival,**  **Month [95% CI]** | **Hazard Ratio**  **[95% CI]** | ***P*-value** |
| --- | --- | --- | --- | --- | --- |
| mGI | < 15 cCAFs | 17 | Not defined |  |  |
|  | ≥ 15 cCAFs | 6 | 9.5 [3.698-15.292] | 1.809 [0.521-6.280] | 0.290 |
| mPDAC | < 15 cCAFs | 9 | 14.2 [6.055-22.332] |  |  |
|  | ≥ 15 cCAFs | 13 | 3.2 [0.801-5.855] | 2.999 [1.168-7.700] | **0.013** |
| Localized PDAC | ≤ 148.2 ng/ml | 22 | 23.5 [7.304-39.678] |  |  |
|  | > 148.2 ng/ml | 7 | 12.6 [not defined] | 6.306 [0.264-150.6] | **0.008** |
| mPDAC | ≤ 148.2 ng/ml | 14 | 5.290 [2.786-7.794] |  |  |
|  | > 148.2 ng/ml | 10 | 1.774 [1.163-2.385] | 3.353 [1.185-9.485] | **0.004** |
| mPDAC | Rest | 12 | 3.910 [1.344-6.475] |  |  |
|  | ≥ 15 cCAFs and  > 148.2 ng/ml | 7 | 1.708 [1.118-2.299] | 3.086 [0.909-10.47] | **0.015** |

**Supplementary Table 3:** Overall survival of the study groups stratified by cCAF and HA plasma levels. Abbr.: cCAF: Circulating cancer-associated fibroblast, HA: Hyaluronan, CI: Confidence interval, mGI: Metastatic gastrointestinal malignancies, mPDAC: Metastatic pancreatic ductal adenocarcinoma.

**Supplementary Table 4:** *Univariate and multivariate Cox analyses for overall survival of the localized PDAC cohort according to clinical characteristics and plasma HA levels*

|  |  | **Univariate analysis** | |  | **Multivariate analysis** | | |
| --- | --- | --- | --- | --- | --- | --- | --- |
| **Variable** | **N** | **HR (95% CI)** | ***P*‑*value*** |  | | **HR (95% CI)** | ***P*-value** |
| HA > 148.2 ng/ml  (vs. ≤ 148.2 ng/ml) | 7/22 | 8.175 [1.306-51.171] | **0.025** |  | | 3.782 [0.554-25.822] | 0.175 |
| Age ≥ 75 years  (vs. < 75 years) | 12/17 | 1.007 [0.237-4.277] | 0.992 |  | |  |  |
| Sex  (Female vs. male) | 18/11 | 4.144 [0.923-18.601] | 0.063 |  | |  |  |
| ECOG 0  (vs. ≥ 1) | 20/9 | 2.120 [0.498-9.021] | 0.309 |  | |  |  |
| UICC III  (vs. I-II) | 10/19 | 6.084 [1.167-31.730] | **0.032** |  | | 4.585 [0.781-26.895] | 0.092 |
| R Status  (R0 vs. R1) | 21/8 | 1.276 [0.298-5.474] | 0.743 |  | |  |  |
| Clavien-Dindo 0-2  (vs. 3-4) | 16/13 | 0.594 [0.138-2.558] | 0.484 |  | |  |  |
| CA19-9 ≥ 500 kU/l  (vs. < 500 kU/l) | 22/6 | 2.054 [0.466-9.045] | 0.341 |  | |  |  |

**Supplementary Table 4:** Univariate and multivariate Cox analyses for overall survival of the localized PDAC cohort according to clinical characteristics and plasma HA levels. Abbr.: HA: Hyaluronan, CI: Confidence interval, mGI: Metastatic gastrointestinal malignancies, mPDAC: Metastatic pancreatic ductal adenocarcinoma, ECOG: Eastern Cooperative Oncology Group, UICC: Union for International Cancer Control, R: Residual tumor, CA19-9: Carbohydrate antigen 19-9.

**Supplementary Table 5:** *Trend of increased biomarkers under progress*

| **Sample ID** | **Sample collection** | **cCAFs** | **CTCs** | **HA [ng/ml]** |
| --- | --- | --- | --- | --- |
| 12340 | Baseline | n.d. | n.d. | 53.21 |
|  | Progress | 18 | 0 | 221.3 |
| 12439 | Baseline | n.d. | n.d. | 20.55 |
|  | Progress | 12 | 0 | 100.52 |
| 12980 | Baseline | 27 | 2 | 45.05 |
|  | Progress | 25 | 12 | 87.92 |
| 12727 | Baseline | 0 | 0 | 31.17 |
|  | Progress | 63 | 1 | 53.21 |

**Supplementary Table 5:** Trend of increased biomarkers under progress. Levels of circulating cancer-associated fibroblasts (cCAFs), circulating tumor cells (CTCs) and hyaluronan (HA) in patients (N=4) with metastatic pancreatic ductal adenocarcinoma (mPDAC) at baseline and at the time of disease progression. Abbr.: n.d.: not determined.

**Figures in ppt slide (supplementary):**

**Supplementary Figure 1:** Representative brightfield images of the primary human pancreatic ductal adenocarcinoma

cancer-associated fibroblast (CAF) cell lines LueCAF11 and LueCAF18 at 10x magnification. The scale bar of 200 µm applies to all pictures.

**Supplementary Figure 2:** Comparison of mean overall CAF recovery rates using Parsortix^TM^, MACS® cell separation (n=6) and the sequential assay (n=8). Y‑axis indicates the mean percentage of recovered DAPI^+^ CMFDA^+^ positive cells.

**Supplementary Figure 3:** Representative images of immunohistochemical stainings of PDAC tissues for FAPα, αSMA and hyaluronan binding protein (HABP). Staining showing (left) high and (right) negative/low expression intensity. The scale bar of 50 µm applies to all pictures.
